# ﻿Notes on the *Pselaphodes* Westwood complex (Coleoptera, Staphylinidae, Pselaphinae) of Hubei, China, with description of a new species and additional faunistic data

**DOI:** 10.3897/zookeys.1206.126696

**Published:** 2024-07-08

**Authors:** Ting Feng, Zi-Wei Yin

**Affiliations:** 1 Shanghai Zoological Park, 2381 Hongqiao Road, Changning District, Shanghai, 200335, China Shanghai Zoological Park Shanghai China; 2 Laboratory of Systematic Entomology, College of Life Sciences, Shanghai Normal University, 100 Guilin Road, Xuhui District, Shanghai 200234, China Shanghai Normal University Shanghai China

**Keywords:** Ant-loving beetle, central China, identification key, taxonomy, Tyrini

## Abstract

The *Pselaphodes* Westwood complex of genera is represented in Hubei Province by four genera and eight species. Recent field work at Wanchaoshan Nature Reserve, Xingshan County revealed a small series of material belonging to this complex. In this paper, we describe *Pselaphodeswanchaoshanus***sp. nov.** and provide new faunistic data for *P.nomurai* Yin, Li & Zhao. A key to the hitherto known members of *Pselaphodes* complex that occur in Hubei is provided to facilitate ready species identification.

## ﻿Introduction

The *Pselaphodes* Westwood complex of genera (sensu Hlaváč 2003) is a speciose group including nine morphologically similar, and probably also phylogenetically close, genera (Yin et al. 2013a) of the tribe Tyrini that are diversified primarily in the Oriental Region. As of 1^st^ May, 2024, 179 species of this complex have been described (Newton 2022). The most diverse genus, *Pselaphodes* Westwood, contains 87 species (e.g., Huang et al. 2018a, 2018b; Huang and Yin 2019, 2020; Yin 2019; Yin and Li 2021), followed by *Labomimus* Sharp with 48 species (e.g., Zhang and Yin 2019; Zhang et al. 2019; Li and Yin 2020; Yin and Li 2021), *Linan* Hlaváč with 17 species (e.g., Yin et al. 2011a; Yin and Li 2013a; Zhang et al. 2018; Zhao et al. 2019), *Lasinus* Sharp with 12 species (Bekchiev et al. 2013; Yin et al. 2014), and the other, smaller genera *Nomuraius* Hlaváč (Huang and Yin 2018), *Paralasinus* Hlaváč & Nomura (Hlaváč and Nomura 2001), *Taiwanophodes* Hlaváč (Bekchiev 2010), *Dayao* Yin, Li & Zhao (Yin et al. 2011b, 2013a), and *Indophodes* Hlaváč (Hlaváč 2003), each comprising no more than five species.

Motivated by Prof. Cong Wei of Northwest A&F University, our team recently had an opportunity to visit a few interesting collecting sites at several nature reserves in Hubei, and we successively obtained a short series of pselaphine beetles (c. 80 specimens). An attempt to identify this material revealed a new species of *Pselaphodes*, which represents the fourth member of the genus in Hubei. Simultaneously collected were two adults of *Pselaphodesnomurai* Yin, Li & Zhao, a well-known species distributed across the Qinling Mountains. This new find is reported in this paper, and a key to aid in the identification of all known species of the *Pselaphodes* complex (nine species) occurring in Hubei is provided.

## ﻿Materials and methods

The materials treated in this paper are deposited in the Insect Collection of Shanghai Normal University (SNUC). The label data of the material is quoted verbatim. Dissected parts were mounted in Euparal on plastic slides pinned with the specimen. The habitus image of the beetle was taken using a Canon EOS R5 camera, equipped with a 5× Mitutoyo M Plan Apo lens, and three 20W UFO LED bulbs (5000 k) were used as the light source. Images of morphological details were produced using a Canon G9 camera mounted to an Olympus CX31 microscope under reflected or transmitted light. Helicon Focus v. 8.2.0 Pro was used for image stacking. All images were modified and grouped into plates using Adobe Photoshop CC 2020.

Measurements were taken as follows: total body length was measured from the anterior margin of the rostrum to the apex of the abdomen; head length was measured from the anterior margin of the rostrum to the head base, excluding the cervical constriction; head width was measured across the eyes; the length of the pronotum was measured along the midline, the width of the pronotum equals the maximum width; the length of the elytra was measured along the suture; the width of the elytra was measured as the maximum width across both elytra; the length of the abdomen is the length of the dorsally exposed part of the abdomen along its midline, the width is the maximum width. The terminology follows Chandler (2001) and Yin (2022). Abdominal tergites and sternites are numbered in Arabic (starting from the first visible segment) and Roman (reflecting true morphological position) numerals, e.g. tergite 1 (IV), or sternite 1 (III). Paired appendages in the description are treated as singular.

## ﻿Taxonomy

### ﻿Key to species of *Pselaphodes* complex occurring in Hubei Province, China (males)

**Table:** 

1	Maxillary palpus small and simple, almost symmetrical, lateral margin of palpomeres 2–4 lacking expansion or projection (Locality: Guanmenshan)	***Lasinussinicus* Bekchiev, Hlaváč & Nomura**
–	Maxillary palpus asymmetrical, lateral margin of palpomeres 2–4 expanded or projected	**2**
2	Vertexal and frontal fovea indistinct or absent	**3**
–	Vertexal and frontal fovea distinct	**4**
3	Frons lacking fovea (Zhang et al. 2018: fig. 1A); antennomere 9 angulate to lateral margin and with small tubercle near apex, 10 simple (Zhang et al. 2018: fig. 2A); mesotibia greatly curved at middle (Zhang et al. 2018: fig. 2G); aedeagus with elongate parameres (Zhang et al. 2018: fig. 2J–L) (Locality: Xingdoushan, Changtanhe, Huangjindong)	***Linanarcitibialis* Zhang, Li & Yin**
–	Frons with small, indistinct fovea (Zhang et al. 2018: fig. 10A); antennomere 9 broadened through length, 10 greatly transverse (Yin et al. 2011a: fig. 11; Zhang et al. 2018: fig. 10B); mesotibia moderately curved at middle; aedeagus with greatly broadened parameres (Yin et al. 2011a: figs 35, 36; Zhang et al. 2018: fig. 10K, J) (Locality: Xingdoushan)	***Linanmegalobus* Yin & Li, 2011**
4	Setose metaventral fovea present; postgenae broadly expanded laterally (Yin and Li 2012: fig. 1B) (Locality: Dabashan)	***Labomimusdabashanus* Yin & Li**
–	Setose metaventral fovea absent; postgenae convergent posteriorly	**5**
5	Body length 2.2–2.4 mm; antennal club simple, lacking modifications (Yin et al. 2011c: fig. 22) (Locality: near Xueluozhai)	***Pselaphodesparvus* Yin, Li & Zhao**
–	Body length no less than 3.0 mm; antennal club modified	**6**
6	Antennomere 9 subcylindrical, with disc-like projection near apex, antennomere 10 and 11 lacking modifications	**7**
–	Antennomere 9 subtriangular, lacking disc-like projection, antennomere 10 and 11 greatly modified	**8**
7	Metaventral processes in lateral view straight, broadened apically and with broad notch at apex (Yin et al. 2013a: fig. 9C); medial lobe of aedeagus extended, with narrowed apex (Yin et al. 2013a: fig. 9J) (Locality: Dabieshan)	***Pselaphodesanhuianus* Yin & Li**
–	Metaventral processes in lateral view curved at middle, narrowing apically and with pointed apex (Yin et al. 2013a: fig. 15C); medial lobe of aedeagus extended and broadening toward apex (Yin et al. 2013a: fig. 15J) (Locality: Dabieshan)	***Pselaphodeslongilobus* Yin & Li**
8	Antennomere 9 subtriangular, lacking large projection (Fig. 1D), 10 round-subquadrate, transverse, dorsal surface broadly impressed (Fig. 1D); metaventral process in lateral view narrowed at apex (Fig. 1E) (Locality: Wanchaoshan)	***Pselaphodeswanchaoshanus* sp. nov.**
–	Antennomere 9 subtriangular, greatly projected on inner apical margin (Yin et al. 2010: fig. 92), 10 elongately oblique, mesal surface impressed (Yin et al. 2010: fig. 92); metaventral process in lateral view roundly broadened at apex (Yin et al. 2010: fig. 79) (Locality: Dabashan, Wanchaoshan)	***Pselaphodesnomurai* Yin, Li & Zhao**

#### ﻿ Pselaphodes nomurai

Taxon classificationAnimaliaColeopteraStaphylinidae

Yin, Li & Zhao, 2010

F454F21B-9ED9-5FC9-9522-19AFB44E5DD7

Pselaphodes nomurai Yin, Li & Zhao, 2010: 21; Yin et al. 2012 (key); Yin and Li 2012 (distribution); Yin et al. 2013b (distribution). Type locality: China, Shaanxi Prov., Foping County (33°31'28"N, 107°59'26"E), elev. 1,250–1,400 m.

##### Additional material examined

**(2 specimens).** 2 ♂♂, “China: Hubei, Xingshan County, Wanchaoshan N. R., 31.3217°N, 110.4906°E, 1700 m, 19.viii.2023, Guo-Hao Wei leg., 湖北兴山县万朝山保护区, 魏国豪采” (SNUC).

##### Distribution.

China: Henan, Shaanxi, Hubei, Chongqing, Sichuan. New distributional record in Hubei.

##### Comments.

These two males can be readily identified as *P.nomurai* by the characteristic form of the male antennal club, the apically broadened metaventral processes, and the aedeagus with an extended, apically truncate median lobe. In comparison to that of the type locality, the Wanchaoshan population has the inner apical margin of male antennomere 10 being greatly protruding to level above the mesal projection of antennomere 9.

#### ﻿ Pselaphodes wanchaoshanus

Taxon classificationAnimaliaColeopteraStaphylinidae

Feng & Yin sp. nov.

7715F79A-CC7A-5EDE-A2A9-042A8FE92EA8

https://zoobank.org/E48B3C8B-AE9F-4B8F-B898-44DBD418707F

**Figure 1.**
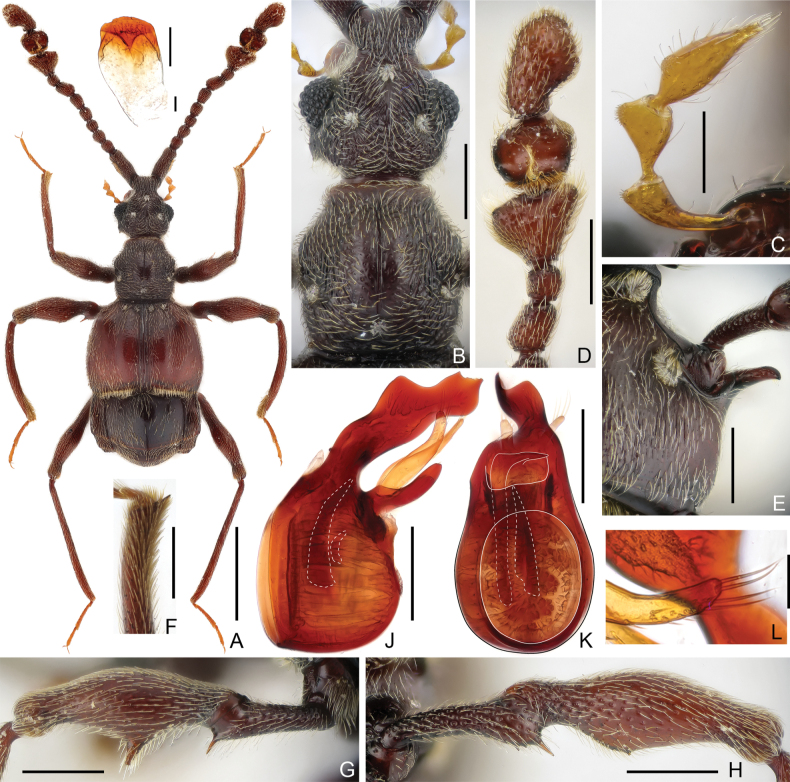
Morphological characters of *Pselaphodeswanchaoshanus* sp. nov. **A** dorsal habitus **B** head dorsum and pronotum **C** maxillary palpus **D** antennal club **E** metaventral process, lateral **F** apex of protibia **G** protrochanter and profemur **H** mesotrochanter and mesofemur **I** sternite 7 (IX) **J, K** aedeagus, lateral (J) and ventral (K) **L** apex of paramere. Scale bars: 1.0 mm (**A**); 0.3 mm (**B, D, E, G, H**); 0.2 mm (**C, F, J, K**); 0.1 mm (**I, L**).

Fig. 1 Chinese common name: 万朝山长角蚁甲

##### Type material

**(1 specimen). *Holotype***: China: ♂, “China: Hubei, Xingshan County, Wanchaoshan N. R., 31.3217°N, 110.4906°E, 1700 m, 19.viii.2023, Guo-Hao Wei leg., 湖北兴山县万朝山保护区, 魏国豪采” (SNUC).

##### Diagnosis.

**Male.** Body length approximately 3.2 mm. Vertex and frontal rostrum with coarse rugose sculpture; maxillary palpomeres 2–4 each roundly protuberant on lateral margin; antenna distinctly clubbed, antennomere 1 with row of dense setae on lateral margin, 7 oblique, 9 subtriangular, 10 broadly impressed on dorsal surface, 11 constricted at base and broadened apically. Center of pronotal disc with large punctures, lacking rugose sculpture, with distinct median longitudinal sulcus. Metaventral process in lateral view short, narrowing apically. Protibia with small apical spur, protrochanter with thin spine, profemur with broad triangular spine; mesotrochanter with one distinct spine and few small denticles, mesofemur with small denticle on ventral margin. Tergite 1 (IV) dorsally more than 3× as long as 2 (V). Aedeagus with broad, extended median lobe, endophallus composed of two long and one short sclerite.

##### Description.

**Male.** Body (Fig. 1A) length 3.16 mm; head, antennae, pronotum and abdomen dark red brown, elytra reddish-brown, tarsi and mouthparts lighter. Dorsal surface of body covered with short pubescence.

Head (Fig. 1B) roundly triangular, subtruncate at base, slightly longer than wide, length 0.69 mm, width across eyes 0.65 mm; vertex with coarse rugose sculpture, with large, setose vertexal foveae (dorsal tentorial pits), with short medio carina between foveae; rostrum prominent anteriorly, covered with rugose sculpture, with large setose frontal fovea; clypeus sharply descending, its anterior margin carinate and moderately raised. Venter with small, widely separated gular foveae (posterior tentorial piss) in broad and deep impression, lacking median carina. Eyes greatly prominent, each composed of approximately 55 ommatidia. Maxillary palpus (Fig. 1C) four-segmented, palpomere 1 minute, 2 pedunculate in basal half and broadening apically, 3 with short stem at base, apical part broadened and subtriangular, 4 subfusiform, elongate, with elongate apical palpal cone; 2–4 each roundly expanded on lateral margin, with short, dense setae at apex of each expansion. Antenna elongate, length 2.35 mm, with modified antennomeres 7 and 9–11 and distinct club (Fig. 1D); antennomere 1 long and thick, subcylindrical, lateral margin with row of dense, short golden setae, 2–6 each submoniliform, of similar width, with 6 slightly longer than 2–5, 7 oblique, longer than 6, 8 shortest, 9 greatly enlarged, subtriangular, 10 broad, narrower than 9, broadly impressed on dorsal surface and with longitudinal row of setae at middle of impression, 11 asymmetric, constricted for basal 1/5, then obliquely broadening to apex.

Pronotum (Fig. 1B) slightly longer than wide, length 0.69 mm, width 0.66 mm, widest at approximately apical 1/3, sides subparallel posterior to broadest point and convergent apically, with almost straight anterior and posterior margin; disc moderately convex, central portion smooth and with large punctures, rest portion with coarse, rugose sculpture; with distinct median longitudinal sulcus and large, setose median and lateral antebasal foveae. Prosternum with basisternal (precoxal) portion at middle shorter than procoxal rests; with setose lateral procoxal foveae; hypomera fused with sternum, smooth, lacking hypomeral grooves and carinae.

Elytra subquadrate, much broader than long, length 0.91 mm, width 1.26 mm, length/width 0.72; each elytron with two large, setose basal foveae; with complete sutural striae and broad longitudinal discal impressions; humeri roundly prominent, lacking subhumeral foveae or marginal striae; posterior margin with row of dense setae. Metathoracic wings fully developed.

Mesoventrite short, laterally fused with metaventrite; mesanepisterna and anterior region of mesoventrite forming transverse prepectus, posteriorly mesoventrite smoothly broadening, with lateral margins moderately diverging; median mesoventral foveae broadly separated in setose transverse impression, lateral mesoventral foveae large and setose, not forked (straight) internally; intercoxal process blunt and short. Metaventrite weakly impressed at middle, with pair of elongate metaventral processes, laterally each process (Fig. 1E) narrowing toward apex; large, setose lateral mesocoxal foveae present; posterior margin with narrow slit in middle.

Legs elongate; protibia (Fig. 1F) with small apical spur, protrochanter (Fig. 1G) with thin, acute spine and profemur (Fig. 1G) with large, broad triangular spine on ventral margin; mesotrochanter (Fig. 1H) with one long and acute and few small denticles, and mesofemur (Fig. 1H) with single small denticle on ventral margin; hind leg simple.

Abdomen widest at lateral margins of tergite 1 (IV), length 1.08 mm, width 1.18 mm, with well-developed paratergites 1–4. Tergite 1 (IV) in dorsal view approximately 3.3× as long as 2 (V), with broad, setose basal impression, discal carinae broadly separated, extending posterior for approximately 1/4 tergal length, tergite 2 (V) and 3 (VI) each short, subequal in length, 4 (VII) longer than 3, posterior margin angularly convex at middle, 2–4 each with one pair of basolateral foveae, 5 (VIII) transverse, posterior margin narrowed and roundly emarginate at middle. Sternite 2 (IV) longest, with densely setose basal sulcus and one pair of mediobasal and basolateral foveae, 3 (V) to 5 (VII) at middle successively shorter, each with one pair of small basolateral foveae, 6 (VIII) transverse, posterior margin with small emargination at middle, 7 (IX) (Fig. 1I) elongate, semisclerotized in apical portion and membranous basally.

Aedeagus (Fig. 1J, K) 0.61 mm long, dorso-ventrally asymmetric; median lobe with broad basal capsule and large, oval dorsal diaphragm, apical portion broadened and greatly extended, with narrowed apex; endophallus composed of two elongate and one short sclerite; parameres (Fig. 1L) each elongate, membranous, with five small setae along ventral margin in apical part and four long macrosetae at apex.

**Female.** Unknown.

##### Comparative notes.

This species is placed as a member of the Walkeri group (*sensu*Huang et al. 2018a) based on the asymmetric male antennomeres 7. The subtriangular antennomeres 9 of the male resemble those of *P.anjiensis* Huang, Li & Yin (Zhejiang), *P.antennarius* Huang, Li & Yin (Guizhou), *P.pseudowalkeri* Yin & Li (Zhejiang, Fujian, Jiangxi), and *Pselaphodeswalkeri* (Sharp) (Zhejiang), but the new species can be readily separated by the broadly impressed antennomeres 10, basally constricted and apically broadened antennomeres 11, moderately long metaventral processes, the spination of the legs, as well as the configuration of the aedeagus. The antennomeres 11 of this species are also similar to those of *P.nomurai*, but the forms of the antennomeres 9 and 10 are quite different.

##### Distribution.

China: Hubei.

##### Etymology.

The species is named after its type locality, i.e., Wanchaoshan.

## Supplementary Material

XML Treatment for Pselaphodes nomurai

XML Treatment for Pselaphodes wanchaoshanus

## ﻿Additional information

### Author ORCIDs

Zi-Wei Yin https://orcid.org/0000-0001-6659-9448

### Data availability

All of the data that support the findings of this study are available in the main text.
